# Investigate the Effect of Thawing Process on the Self-Assembly of Silk Protein for Tissue Applications

**DOI:** 10.1155/2017/4263762

**Published:** 2017-03-07

**Authors:** Hiep Thi Nguyen, Hien Thu Luong, Hai Dai Nguyen, Hien Anh Tran, Khon Chan Huynh, Toi Van Vo

**Affiliations:** ^1^Tissue Engineering and Regenerative Medicine Laboratory, Department of Biomedical Engineering, International University-Vietnam National University-Ho Chi Minh City (VNU-HCM), Ho Chi Minh City 700000, Vietnam; ^2^Institute of Applied Materials Science, Vietnam Academy of Science and Technology, Ho Chi Minh City 70000, Vietnam

## Abstract

Biological self-assembly is a process in which building blocks autonomously organize to form stable supermolecules of higher order and complexity through domination of weak, noncovalent interactions. For silk protein, the effect of high incubating temperature on the induction of secondary structure and self-assembly was well investigated. However, the effect of freezing and thawing on silk solution has not been studied. The present work aimed to investigate a new all-aqueous process to form 3D porous silk fibroin matrices using a freezing-assisted self-assembly method. This study proposes an experimental investigation and optimization of environmental parameters for the self-assembly process such as freezing temperature, thawing process, and concentration of silk solution. The optical images demonstrated the possibility and potential of −80ST48 treatment to initialize the self-assembly of silk fibroin as well as controllably fabricate a porous scaffold. Moreover, the micrograph images illustrate the assembly of silk protein chain in 7 days under the treatment of −80ST48 process. The surface morphology characterization proved that this method could control the pore size of porous scaffolds by control of the concentration of silk solution. The animal test showed the support of silk scaffold for cell adhesion and proliferation, as well as the cell migration process in the 3D implantable scaffold.

## 1. Introduction

Self-assembly is a process by which components spontaneously form a well-defined stable structure under several certain conditions. This process is scattered in natural systems (e.g., secondary and higher order structures of protein) and is investigated for engineered systems as a promising method to fabricate supermolecular architecture and harness biomaterial properties from the bottom up [1–4]. The potential of self-assembly is represented by its ability to form far-beyond-molecules structures with controllable properties and the creation of functional complexes, which are dependent on both material features and process assigned conditions.

In nature, silk is one of the extraordinary examples of self-assembly into fiber with exceptional mechanical strength and elasticity. In tissue engineering, the number of studies focusing on silk protein investigation and silk-based material production based on its natural self-assembly property is remarkable [5–8]. Those investigations indicate that silk fibroin solution, which contains mainly random coil structures, is engineered and self-assembled into *β*-sheets and *α*-helix structure by the addition of alcohol or steam treatment [1, 9]. However, alcohol and steam treatments could lead to uncontrollable crystallization and brittle material [10]. On the other hand, it is proven that environmental parameters of silk solution such as temperature, concentration, and pH level are critical for the self-assembly process [11, 12]. There were several studies focusing on the effect of temperature in different phases of fabrication on silk protein self-assembly [13]. For example, Kweon et al. showed that heat treatment on silk-based films could induce secondary conformation transformation [14]. The group of Putthanarat [15] and Zhong [16] pointed out that the drying and incubation temperature had a notable effect on the morphology of silk fibroin nanostructures. Other researches which investigated the effect of freezing temperature before lyophilization and alcohol treatment were also reported [17, 18].

For tissue engineering, porous three-dimensional scaffolds (3D scaffolds), providing a framework with mechanical strength necessary for cellular adherence, proliferation, migration, ECM formation, and nutrients transportation before the tissue develops enough to support itself, play an important role in manipulating cell functions in the regenerative medicine field [19–21]. Several methods have been developed to generate 3D silk porous scaffolds, including fiber bonding, solvent casting/particulate leaching (porogen leaching), compression molding, extrusion, freeze-drying, phase separation, gas forming, 3D printing, and electrospinning [22–24]. In those methods, freeze-drying has been well developed to fabricate porous silk fibroin scaffolds. However, to our knowledge, silk self-assembly process and water-stable 3D scaffold fabrication via freezing method without lyophilization and alcohol or water vapor annealing have not been studied.

In the present study, we focused on roles of thermal treatment, especially freezing temperature, in controlling the scaffold-forming process via the silk fibroin self-assembly and influencing the scaffolds' structure and bioproperties. At the same time, the goal of the present study was to investigate an all-aqueous fabrication process of porous silk scaffold avoiding organic solvents and harsh chemicals, beneficial to the future use of these material systems in cell/growth factor delivery. In particular, silk nanofibrous scaffolds were prepared under an all-aqueous process and under −20°C and −80°C conditions before slowly and steadily increasing the temperature to 8°C. Three factors, that is, thawing rate, freezing temperature, and solution concentration, were studied to identify their effects on silk protein self-assembly, scaffold formation, pore size, and pore uniformity. Morphology and pore geometry of thermal-induced scaffold were observed by a light microscope. Secondary structure, focused on the presence of *β*-sheets, was clarified by the deconvolution of FT-IR spectra. Animal test was employed to observe cell migration and proliferation into the scaffolds.

## 2. Materials and Methods

### 2.1. Materials

*Bombyx mori* (*B. mori*) silkworm cocoons were supplied by Viet Silk Ltd. Co., Lam Dong, Vietnam. The supplied cocoons were in fresh status and did not undergo under any thermal, mechanical, or chemical treatment. Sodium hydroxide (NaOH), sodium carbonate (Na_2_CO_3_), citric acid (C_6_H_8_O_7_), calcium chloride anhydrous (CaCl_2_), and ethanol (C_2_H_5_OH) were purchased from Xilong Chemical, Ltd., China. Naphthol blue black dye was purchased from Hoang Ha Co., Ltd., Vietnam. Dialysis membrane (Spectra/Por 4, Standard RC Tubing, MWCO: 12–14 kDa) was purchased from Spectrum Laboratories, Inc., United States. The in vivo experiment was performed on Swiss Albino mice, about 30 g, provided by Pasteur Institute, Ho Chi Minh City, Vietnam. Hematoxylin and eosin (H&E) staining chemicals, including hematoxylin, eosin, and xylene, were purchased from Sigma-Aldrich, Inc., Germany.

#### 2.1.1. Sample Preparation

*Silk Solution Preparation*. Silk fibroin was prepared from* Bombyx mori* silk fiber by a stepwise purification method with fiber degumming, dissolution, and dialysis. Briefly, raw cocoons were boiled in degumming solution of 0.06 M Na_2_CO_3_ and natural soap solution (pH = 11.6) at 80°C for 30 minutes, followed by distilled water at 70°C to remove sericin. Degummed silk fibers were dissolved in Ajisawa's reagent solution of CaCl_2_, C_2_H_5_OH, and distilled water with mole ratio of 1 : 2 : 8 at 80°C for 1 hour before being dialyzed using a dialysis tube (MWCO: 12–14 kDa) for 3 days at 4°C [25]. The final concentration of aqueous silk solution was determined by weighing the remaining solid after drying (*n* = 3, SD ≤ 0.13). The solution was diluted into 5 concentrations with 1.2% step decrement, from 6.0% to 1.2%, for next experiments.

**Figure 1:**
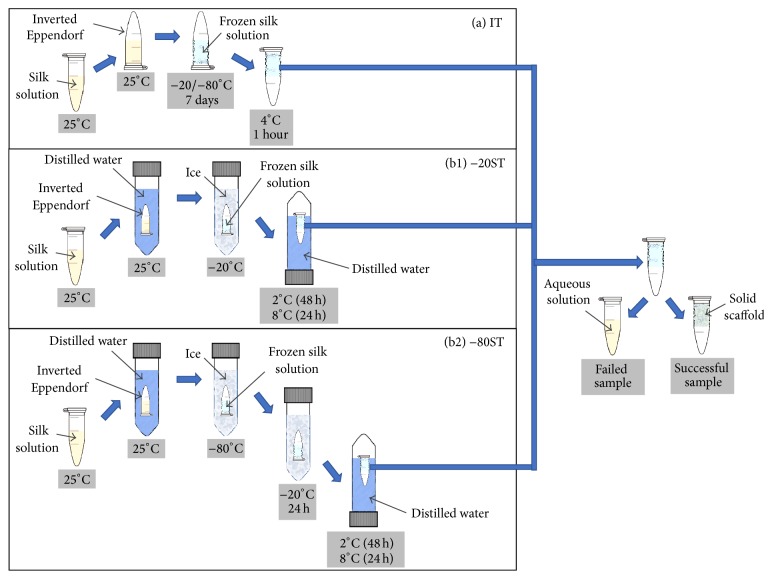
Illustration of the thawing process: (a) IT designed for immediate thawing in 1 hour and (b) ST to control and decelerate the thawing process into 24 (ST24) and 48 (ST48) hours using water as a temperature regulator. While the IT process was the same for −20°C and −80°C frozen samples, the −80°C samples (b2) in ST process would be moved to and stocked at −20°C for 24 hours before being increased to 2°C and 8°C to be thawed (b1).

*Designation of the Thawing Process*. To investigate the effects of thawing rate on the self-assembly of silk protein, two experimental thawing processes were designed and named immediate thawing (IT) (Figure 1(a)) and slow thawing (ST) (Figure 1(b)). In IT, silk solution was added to Eppendorfs before being frozen at −20°C/−80°C. After 7 days, the Eppendorfs were inverted and incubated at 8°C. Iced silk solution would be completely thawed in 1 hour (both −20°C and −80°C samples, called −20IT and −80IT, resp.). In ST, the silk-containing Eppendorfs were put into a 50 mL falcon containing 30 mL distilled water, before being frozen at −20°C/−80°C. After 7 days, samples at −20°C (−20ST) would be inverted and incubated at 8°C and 2°C and the samples were completely thawed after 24 (−20ST24) and 48 (−20ST48) hours, respectively, while the samples at −80°C (−80ST) would be moved to and stocked at −20°C for the first 24 hours before being increased to 8°C and 2°C to be thawed for the next 24 (−80ST24) and 48 (−80ST48) hours. In short, the difference among these samples was the thawing time, which was 1 hour (IT), 24 hours (ST24), and 48 hours (ST48). All three groups were kept incubated at 8°C for 24 hours. The designed thawing processes were used in the following experiments. Control group was the silk solution which did not undergo any further treatment. 

*Investigation of the Effect of the Thawing Rate at Different Freezing Temperatures*. Six groups of 6% silk solution were prepared. The first to the sixth group then underwent −20IT, −20ST24, −20ST48, −80IT, −80ST24, and −80ST48 processes, respectively. 

*Investigation of the Effect of the Thawing Rate on Different Silk Solution's Concentrations*. Fifteen groups of silk solution at different concentrations, 1.2%, 2.4%, 3.6%, 4.8%, and 6.0%, were prepared. Every 3 groups with the same concentration underwent the −80IT, −80ST24, and −80ST48 processes, respectively. 

*Investigation of the Effect of the Freezing Temperature on Different Silk Solution's Concentrations*. Ten groups of silk solution at different concentrations, 1.2%, 2.4%, 3.6%, 4.8%, and 6.0%, were prepared. Every two groups with the same concentration underwent the −20ST48 and −80ST48 processes, respectively. 

*Observation of the Self-Assembly Process*. To demonstrate how the scaffold is formed, the prepared 3.6% silk solution was frozen at −80°C for 1, 2, 3, 4, 5, 6, and 7 days before applying the −80ST48 process. Silk protein self-assembly process in 7 days was recorded by a light microscope every 24 hours (Nikon, Eclipse Ti-U series, Japan).

### 2.2. Characterization of Silk Scaffolds

The surface morphology and pore geometry of scaffolds with different concentrations were visualized using a light microscope. Thin porous scaffolds (thickness < 1 mm) were prepared by applying the −80ST48 to silk solution with different concentrations (6.0%, 4.8%, 3.6%, 2.4%, and 1.2%). The average pore sizes of silk scaffolds were also evaluated based on light microscope images using ImageJ software as mentioned in a previous publication [26].

Wettability of porous silk scaffold was measured based on the imaging method (Olympus, OM-D EM-5, Japan). Silk scaffolds (cylinder shape with 0.5 cm diameter) were prepared and freeze-dried. Naphthol blue black dye which is diluted with distilled water was dropped on the surface of silk scaffolds, and the minimum time for the drop to be totally absorbed was digitally measured (29 frames per second recording). The experiment was performed at room temperature.

The secondary structure of silk scaffold was characterized by FT-IR (TENSOR II FT-IR spectrometer, Bruker, Germany) in the frequency range of 400–4000 cm^−1^. FT-IR spectra of the silk scaffolds fabricated by the −80ST48 process were compared with those of the untreated silk solution (called control group) and those of the silk scaffold fabricated by using alcohol (called alcohol-immersed group). The alcohol-immersed scaffold was fabricated by freeze-drying 6.0% silk solution before immersing it in ethanol 80% for 15 minutes and drying at room temperature in line with modified protocols by Terada et al. [27].

### 2.3. Biocompatibility Test

To evaluate the biocompatibility, silk scaffolds (cylinder, 0.9 cm in diameter, 0.5 cm in height) were sterilized with 70% ethanol and washed by immersing in autoclaved PBS 1x solution before being subcutaneously implanted at the dorsal region under general anesthesia and antiseptic conditions. After 3 and 6 weeks, mice were sacrificed, and then the regenerated area with the scaffold was extracted. The extracted sample was fixed by 3% formaldehyde and stained by hematoxylin and eosin (H&E) stain. H&E stained samples were observed by a light microscope (Nikon, Eclipse Ti-U series, Japan).

### 2.4. Statistical Analysis

All data were reported as mean ± standard deviation. Each experiment was replicated three times unless otherwise mentioned. Kruskal-Wallis One-Way Analysis and Dunn's Method were performed. All statistical analyses were executed using SigmaPlot 11.0 (Systat Software, San Jose, CA). Deconvolution was executed using Origin 8.0 (OriginLab Corporation, Northampton, MA).

## 3. Results 

### 3.1. Sample Preparation

**Figure 2:**
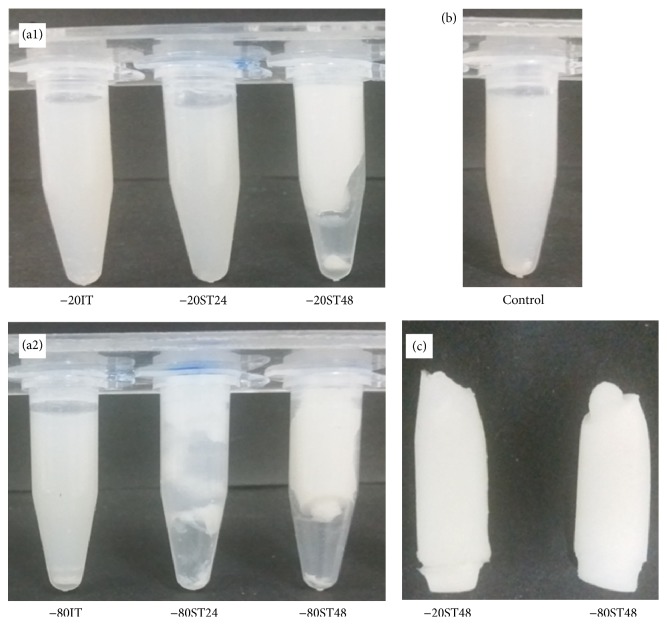
Photographs of Eppendorfs containing samples of IT, ST24, and ST48 to silk scaffold formation, at (a1) −20°C and (a2) −80°C. (b) Silk solution which did not undergo any further treatment as control group. (c) Successfully fabricated scaffolds out of the Eppendorfs, including −20ST48 and −80ST48.

*Investigation of the Effect of the Thawing Rate at Different Freezing Temperatures*. To investigate the effect of thawing on the self-assembly at different freezing temperatures, the designed IT, ST24, and ST48 processes were applied to 6% silk solution at −20°C and −80°C.  Figure 2 shows the photograph of the inverted sample-containing Eppendorfs to compare with the untreated 6% solution as the control. Both −20IT and −80IT samples returned to the initial solution state, which had no visible difference with the control group. White aggregation had occurred with very low density (data not shown). The ST48 groups, including −20ST48 and −80ST48, were all self-assembled into fixed solid forms and easily taken out of the Eppendorf (Figure 2(b)). This result indicates that low rate of thawing process plays a key role in the self-assembly process of silk protein. The 24-hour samples had a noticeable difference between −20ST24 treated and −80ST24 treated groups. While −20°C treated samples were in aqueous state with white aggregations, −80°C treated samples were already self-assembled into a white cloud, but with less stable form than of ST48 group. This evidence slightly implied that lower freezing temperature had a stronger effect on the silk self-assembly process. 

**Figure 3:**
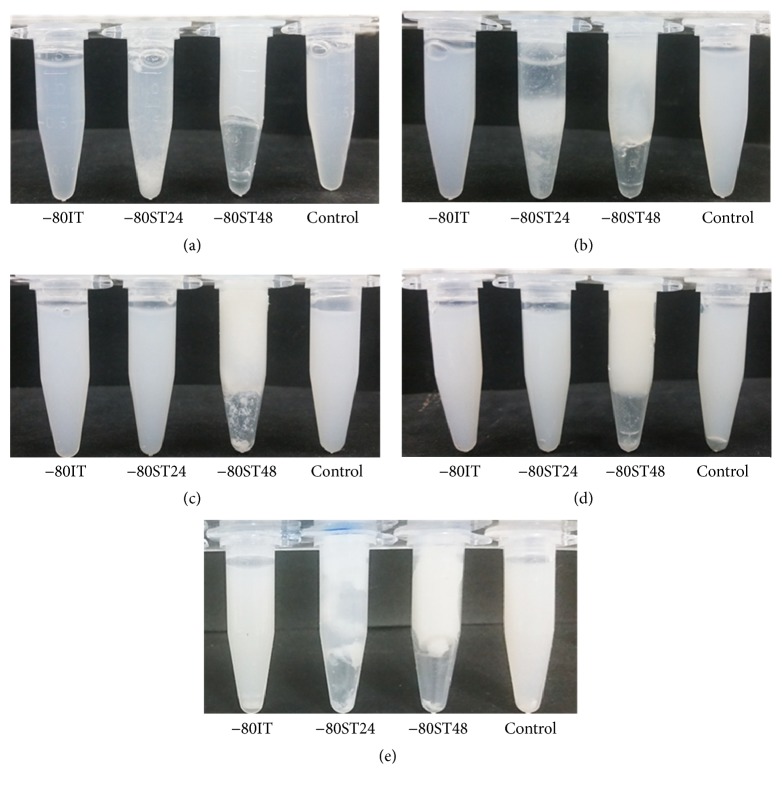
Photographs of Eppendorfs containing samples of IT, ST24, and ST48 to silk scaffold formation, at different concentrations: (a) 1.2%, (b) 2.4%, (c) 3.6%, (d) 4.8%, and (e) 6.0%. Silk solution at each concentration which did not undergo any further treatment as control group.

*Investigation of the Effect of the Thawing Rate on Different Silk Solution's Concentrations*. To investigate the effect of the thawing process on the self-assembly at different silk solution's concentrations, the designed −80IT, −80ST24, and −80ST48 processes were applied to 1.2, 2.4, 3.6, 4.8, and 6.0% silk solutions. The results are presented in Figure 3, in comparison with the untreated silk solutions as the control. After increasing suddenly the temperature from −80°C to 4°C in 1 hour, all −80IT samples at 5 concentrations returned to the initial solution state, with no visible difference to the control group. However, the −80ST48 groups were all self-assembled into solid scaffolds and easily taken out of the Eppendorf. These scaffolds had white color of silk fibroin. In between, −80ST24 groups had inconsistent characters. Some samples returned to the initial solution state, which was not noticeably different from the control group, such as 3.6% and 4.8% samples. Other samples contained white silk aggregations (Figure 3, samples 1.2% and 2.4%) or partly self-assembled into a cloud of silk fibroin but were not stable enough to be removed from the Eppendorf (Figure 3, sample 6.0%). This result indicates that the thawing rate was important for the self-assembly process, no matter at which concentration. The suitable thawing process for successful silk protein self-assembly observed from this and previous tests was ST48. 

**Figure 4:**
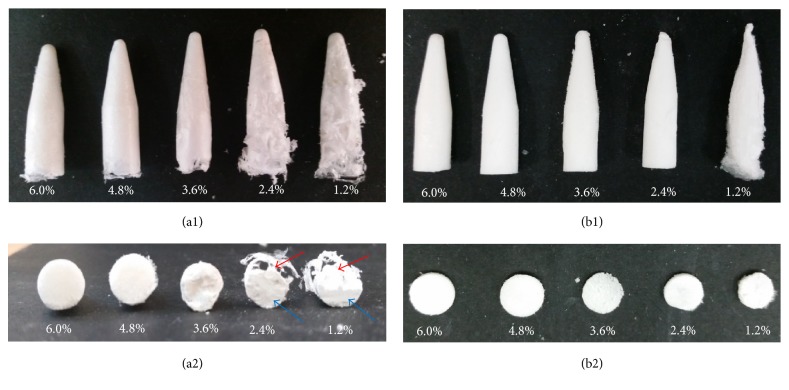
Morphology of (1) silk scaffolds at different concentrations and (2) their cross sections that formed by (a) −20ST48 and (b) −80ST48 processes. The concentration range included 1.2%, 2.4%, 3.6%, 4.8%, and 6.0%.

*Investigation of the Effect of the Freezing Temperature on Different Silk Solution's Concentrations*. The ST48 was indicated as the suitable process for silk assembly that occurred at different freezing temperatures and silk solution's concentrations. The different concentrated scaffolds successfully prepared by −20ST48 and −80ST48 processes are shown in Figure 4 to indicate the effect of freezing temperature on morphology of silk scaffold. There was no significant difference between scaffolds treated at −20°C and −80°C in groups of 6.0, 4.8, and 3.6% solutions. However, for the two lowest concentrated solutions, 1.2% and 2.4%, while scaffolds that were frozen at −80°C were uniformly distributed, −20°C groups were hollow on one side (red arrow) and dense on the other side (blue arrow). The results indicated that freezing temperature could have some visible effects on the scaffold's morphology of low concentrated samples. The −80ST48 was proven to be suitable for fabrication of uniform silk scaffold.

**Figure 5:**
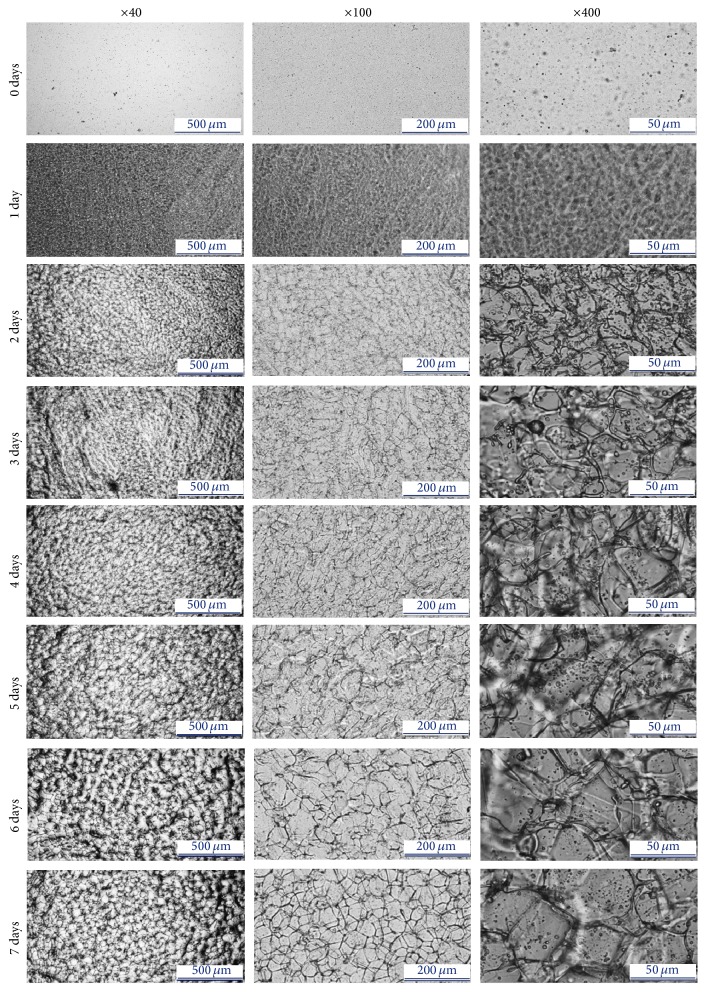
Demonstration of the self-assembly process of silk protein; the prepared 3.6% silk solution was frozen at −80°C for 1, 2, 3, 4, 5, 6, and 7 days before applying the −80ST48 process and recorded by a light microscope every 24 hours, at ×40, ×200, and ×400 magnifications.

*Observation of the Self-Assembly Process*. To observe the formation of silk scaffold via self-assembly process, the morphology of 3.6% silk scaffold which treated the −80ST48 process with different freezing time was observed by a light microscope and represented in Figure 5. Results show that, before any thermal treatment (Figure 5, day 0), the solution contained silk particles, which confirmed the establishment of the initial structure and was available to self-assembly into stable nanofilaments [1]. After being frozen for 2 days, the silk fibroin in the solution subsequently grew and became visible under the light microscope (Figure 5, day 2). These fibroins were short, mostly smaller than 50 *µ*m, and randomly arranged with not much observed linkage among them. Silk fibroins slowly elongated and increased the interactions to form a fibroin network on day 3 to day 5. A porous stable structure formed after freezing for 6 days, which was a porous silk scaffold (Figure 5, days 6 and 7).

### 3.2. Characterization of Silk Scaffolds

**Figure 6:**
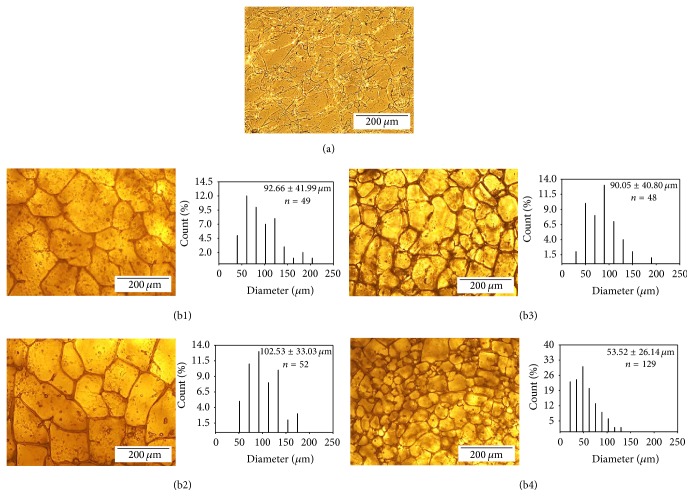
(a) Surface micrograph of 1.2% silk sample which had not formed a stable pore system yet after −80ST48 treatment. (b) Surface's optical micrograph and statistically estimated pore size distribution of manufactured −80ST48 silk scaffolds at (b1) 2.4%, (b2) 3.6%, (b3) 4.8%, and (b4) 6.0%. The statistic figures show the sample size (*n*), the histogram of pore's diameter distribution of 4 samples in the range of 0 *μ*m to 500 *μ*m, and the average pore size as size ± standard deviation.

*Morphology Observation*. Optical micrographs of surface and estimated pore size distribution of manufactured −80ST48 scaffolds at different concentrations were presented in Figure 6. The result indicates that concentration affected the formation of the porous structure and the pore size. For the 1.2% sample, protein chains aggregated to form visible silk fibroins, which forked and connected to each other. However, a structure of stable pore system had not been formed in the 1.2% sample yet. Meanwhile, in 2.4%–6.0% groups, the pore system was clearly observed and distributed from 48.3 to 185.8 *µ*m. Figure 6 demonstrates that the higher concentrated scaffold had smaller pores than the other ones. The average pore sizes of 6.0%, 4.8%, 3.6%, and 2.4% scaffolds were 53.52 ± 26.14, 90.05 ± 40.80, 102.53 ± 33.03, and 92.66 ± 41.99 *µ*m, respectively. 95.35% of the pores of the 6.0% sample were smaller than 100 *µ*m. However, 48.08% and 36.73% of the pores of the 3.6% group and 2.4% group, respectively, are higher than 100 *µ*m. The Kruskal-Wallis One-Way Analysis and Dunn's Method were performed to analyze the difference of the pore size in this group of four. The result indicated that there was no significant difference in pore size between the 2 lowest concentration groups, 2.4%, and 3.6%. Nonetheless, other comparisons (2.4% versus 4.8%, 2.4% versus 6.0%, 3.6% versus 4.8%, 3.6% versus 6.0%, and 4.8% versus 6.0%) showed significant differences much larger than expected by chance or randomness of sampling. In this case, this statistic result indicated that the concentration of silk solution had a significant effect on pore's size distribution, especially to high concentrated solution. However, for the solution that was lower than 2.4% in concentration, the effect of concentration on pore's size distribution was not remarkable. 

**Figure 7:**
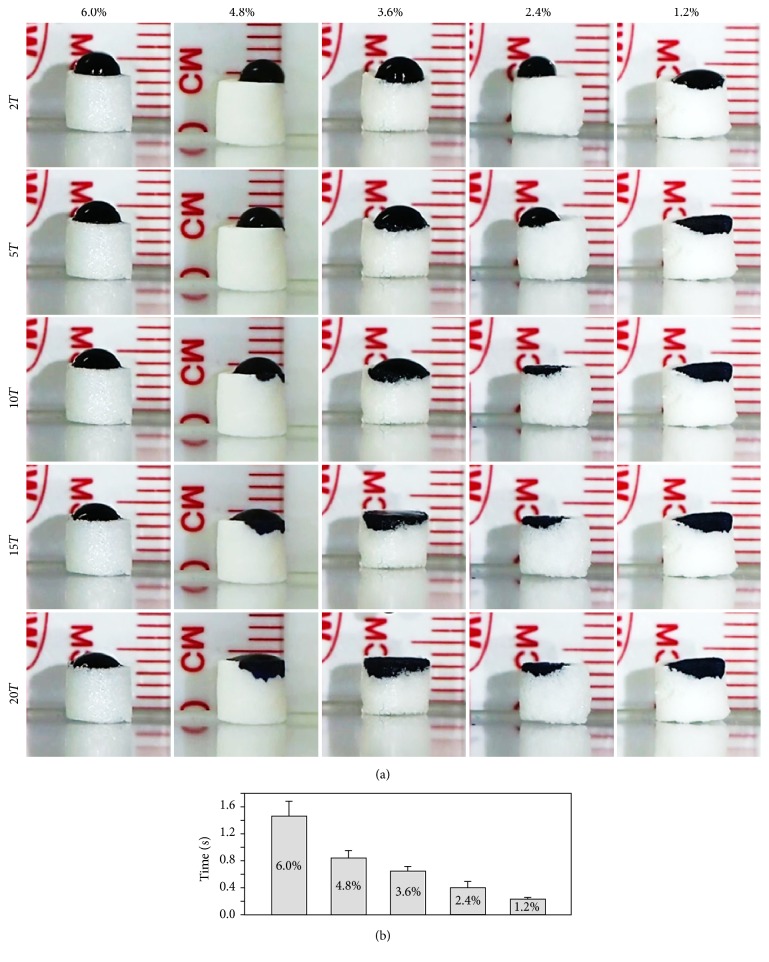
(a) Optical photographs of the stained water drop on 1.2%, 2.4%, 3.6%, 4.8%, and 6.0% concentrated −80ST48 scaffolds after 2*T*, 5*T*, 10*T*, 15*T*, and 20*T* with *T* = 1/29 (about 0.034) seconds. (b) Times for the water drop fully absorbed in 1.2%, 2.4%, 3.6%, 4.8%, and 6.0% concentrated −80ST48 scaffolds were averaged and presented with standard deviation.

**Figure 8:**
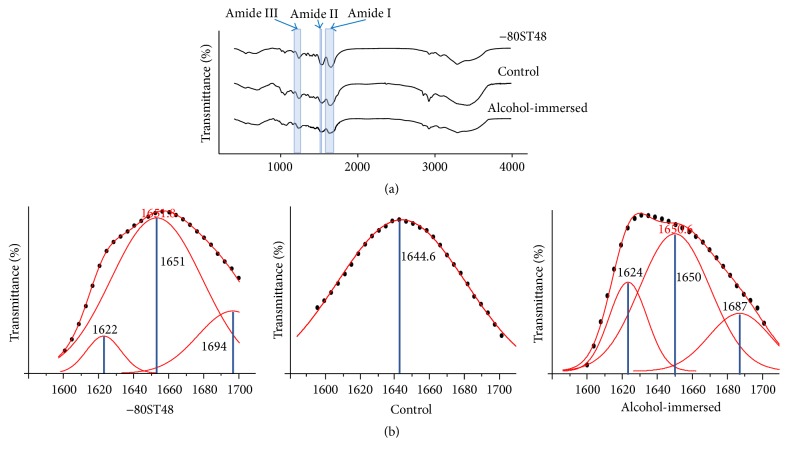
(a) FT-IR spectra of −80ST48, alcohol-immersed, and control scaffold in the range of 400–4000 cm^−1^, with their characteristic peaks of amide I, amide II, and amide III. (b) The deconvolution analysis of amide I's FT-IR spectra of −80ST48 scaffold, compared to the alcohol-immersed scaffold and the control. The black dots were the real measured FT-IR peaks. The red lines represented the component peaks calculated by the deconvolution method, and the lines with black dots were the sum of the calculated component peaks while the blue line indicated the position of each component peak.

*Wettability Test*. For the assessment of pore interconnection of the −80ST48 scaffold at different concentrations, water absorbing process of the scaffold was measured and the optical photographs of water droplets were represented in Figure 7. During the water wettability test, the water drops were fully absorbed by all five concentrations, indicating the water-absorbent structures of −80ST48 scaffold. The high surface wettability is believed to be associated with enhanced protein adsorption and is believed to encourage cell adhesion and spreading [28]. Moreover, the result indicated that the water absorbance of silk scaffold was faster for the lower concentration scaffolds. However, because the exact contact angle could not be reliably assessed (i.e., apparent contact angle of 0°), the time for the water drop to be fully absorbed was recorded, averaged, and shown in Figure 8. 6.0, 4.8, 3.6, 2.4, and 1.2% samples took about 1.46 ± 0.22, 0.84 ± 0.11, 0.64 ± 0.07, 0.4 ± 0.09, and 0.23 ± 0.02 seconds, respectively, to absorb totally the drop, which implied that water was more obstructed by higher concentration samples. 

*FT-IR Spectra*. FT-IR in range of 400–4000 cm^−1^ was used to characterize structure of the −80ST48 silk scaffolds compared to the alcohol-immersed scaffold and untreated silk solution as the control. Silk fibroin's characteristic bands are identified as amide I, amide II, and amide III as shown in Figure 8 [29, 30]. Amide III bands (about 1220–1300 cm^−1^) were the coupled peak of the main C–N stretching and the –N–H in-plane bending vibration, while amide I (about 1600–1700 cm^−1^) and amide II (about 1520–1550 cm^−1^) were the peaks of the carbonyl stretching of –C–O– and the combined peak of the main N–H in-plane bending and the C–H stretching vibration, respectively. Amide I is useful for the analysis of the secondary structure of the proteins, such as *α*-helices, *β*-sheets, and random coils [31, 32]. Among them, the *β*-sheets structure, which contains the strong hydrogen bonds, was proven to enhance the stability, increase the mechanical strength and toughness, and decrease the degradation rate of silk scaffold [24, 33]. The percent of *β*-sheets among secondary structure content was determined by FT-IR spectrum in the range of amide I, 1600–1700 cm^−1^, to evaluate the stability of the −80ST48 sample. The assignment of adsorption peaks was studied by the deconvolution analysis technique and reviewed by Kaplan's group [32, 34].  Figure 8(b) and Table 1 show the deconvolution analysis of FT-IR spectra of −80ST48 scaffold, compared to the alcohol-immersed scaffold and control group. The coefficient of determination (CoD) was used as a measure of the similarity between real signal and mathematically deconvoluted one. All three examined groups have CoD value larger than 0.99, corresponding to a statistically practical match. To the untreated solution, there was only one broad peak at 1645 cm^−1^ assigned to the random coils and extended chains. The alcohol-immersed sample was self-assembled into strong intermolecular *β*-sheet (1624 cm^−1^, 20.33%) and *β*-turn (1687 cm^−1^, 21.39%) structures. The −80ST48 sample had a lower percentage of strong intermolecular *β*-sheets (1622 cm^−1^, 5.80%). The −80ST48 scaffold also contained a large peak at 1689 cm^−1^ (31.10%) which was characteristic of *β*-turn, weak *β*-sheet, or both structures. The result implies that −80ST48 would be less stable than alcohol-immersed samples.

### 3.3. Biocompatibility

**Figure 9:**
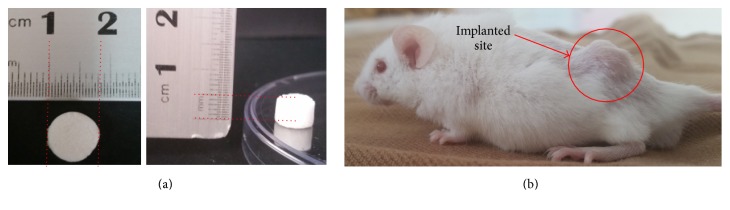
(a) Dimensions of −80ST48 silk scaffolds for in vivo test: cylinder shape with 8 mm diameter and 5 mm height. (b) Subcutaneously implanted sites on the mice lower back after 10 days (red circle).

To test biocompatibility, 6.0% −80ST48 sample was analyzed by subcutaneous implantation in mice.  Figure 9 shows the prepared scaffold for animal test and the photographs of postimplantation of porous silk scaffold after 10 days. The implanted site's wound was closed, hair regrew, and there was no sign of mucus or unusual color area.

**Figure 10:**
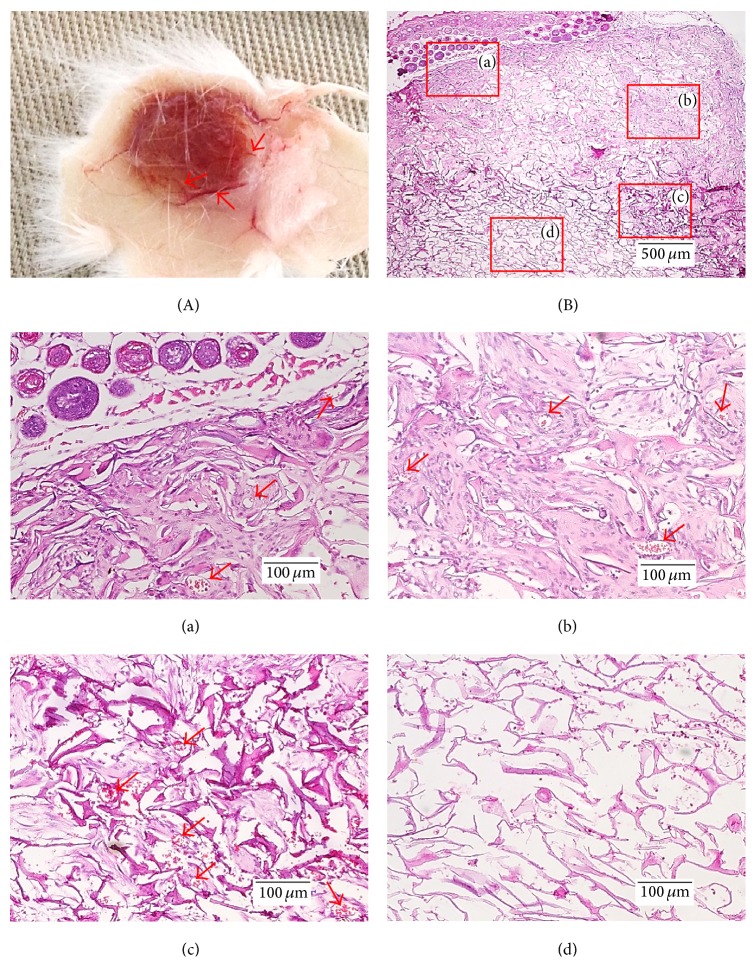
(A) 3-week postimplantation silk sample and (B) its H&E staining figures opened at four positions: from the interface layer (a, b) to the middle of the scaffold (c, d). The red arrows indicate the capillaries on and inside the implanted scaffold.

**Figure 11:**
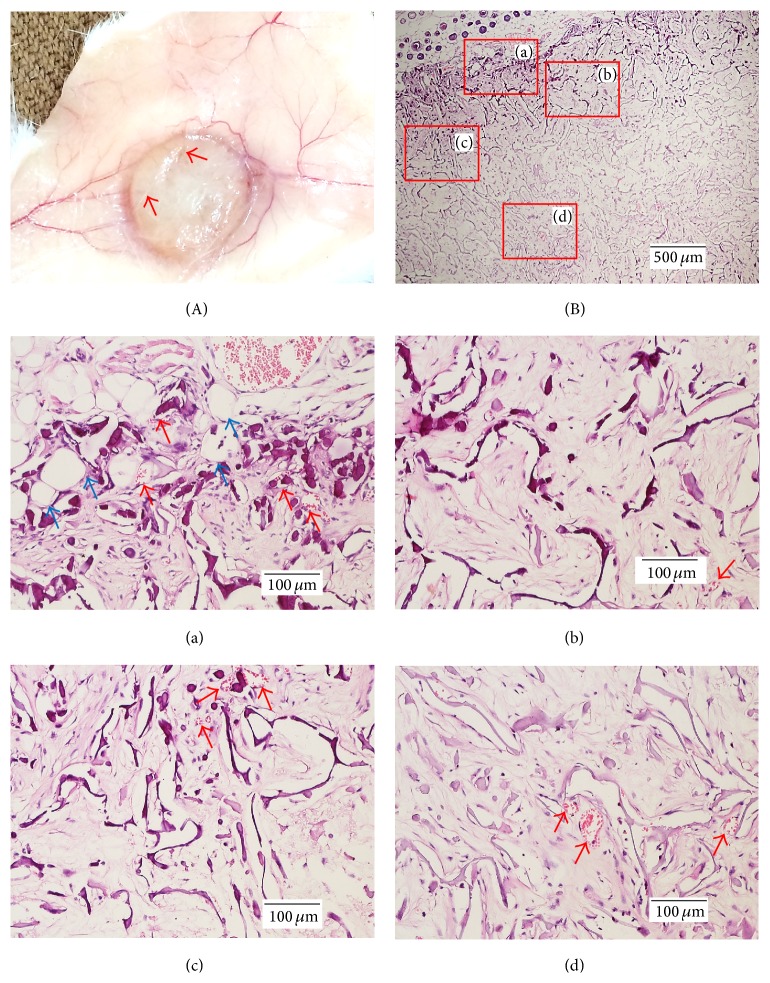
(A) 6-week postimplantation silk sample and (B) its H&E staining figures opened at four positions: from the interface layer (a, b) to the middle of the scaffold (c, d). The red arrows indicate the capillaries on and inside the implanted scaffold.

Figures 10 and 11 represent the extracted −80ST48 samples at 3 and 6 weeks after implantation and their H&E stained histological sections which opened at 4 positions from the outside to the middle of the scaffold to demonstrate the proliferation and migration of the cell inside the silk scaffold. Small capillaries which grew on the surface of the implanted silk scaffold were observed on the postextracted image in Figure 11 and indicated by red arrows. These capillaries transported oxygen, nutrients, and numerous cell types to the implanted scaffold to regenerate the affected sites. Furthermore, they also provided necessary soluble factors such as growth factors, hormones, and cytokines to increase cell migration, ECM formation, and tissue regeneration [35]. The capillary formation on and inside the scaffold was also confirmed by H&E staining images (red arrows). After 3 weeks, the −80ST48 scaffolds showed prominent infiltration and ingrowth of host cell in the interface of implanted sample and the host's skin (Figures 10(a) and 10(b)). Notably, moving along to the middle of the sample (Figures 10(a) and 10(b)to 10(c) and 10(d)) resulted in the decrease of cell infiltration and increase of accumulation of inflammatory cells. In some middle areas in particular, as in Figure 10(d), there was no host cell presence. H&E staining figures demonstrate the cell migration gradient into the 3D scaffold, from the surface to the center direction.

Besides, at 6 weeks after operation, there were a remarkable number of capillaries that grew toward the sample (Figure 11). However, because the surface of the extracted scaffold was covered by a new tissue layer, the surface's capillaries were not able to be observed. The neovascularization was then confirmed by histology result (red arrows). The H&E staining images of 6-week implanted sample also show that the whole scaffold, including the middle, was covered with a soft tissue with little vascularization, and the inflammation reaction of the tissue was not so obvious as before. Fibroblasts ingrowth inside the scaffold's pores was connected to creation of a continuous tissue, which illustrated the interconnection of the scaffold. Moreover, the subcutaneous fat cells began to spread into the interface area of the silk scaffold (Figure 11(a), blue arrows) which demonstrates the degradation of the sample as well as the regeneration of mice's body after 6 weeks.

## 4. Discussion 

One remarkable candidate for natural material is silk protein extracted from* B. mori* silk cocoon, which has been effectively used in many biomedical applications because of its impressive mechanical properties, water permeability, biocompatibility, and biodegradability [5, 6, 8]. Silk solution was previously used in various forms such as 3D porous scaffold, electrospun fibers, thin film, hydrogel, and microsphere [36–43]; most works have been done on self-assembly processes.

Self-assembly occurs when molecules interact through noncovalent interactions, such as hydrogen bonding, hydrophobic forces, van der Waals forces, and electrostatic effects. Although these bonds are weak, the collective interactions can result in a stable structure [1–4]. Fabrication of silk scaffold from silk solution via the self-assembly process was widely studied in previous research [1, 11–13, 44, 45], in which increase in the *β*-sheet content of silk is facilitated by accelerating the process of fibril formation, enhancing then the impressive physical properties as well as thermal stability of silk scaffold. The interaction of the components with their environment, which could be controlled by other factors such as temperature or pH level, can strongly influence the course of the process. Putthanarat et al. presented initial observations of the effect of processing temperature on morphology, which exhibit larger grains and larger, more densely packed nanofibrils [15]. The result indicated that the conversion of the amorphous structure to the *β*-sheet structure was affected by incubation temperature, from 20°C to 50°C. Zhong et al. stated that the incubation temperature (from 25°C to 75°C) could affect the morphology and conformation of silk protein [16]. These and other numerous reports presented some initial observations of high-temperature treatment effect on *β*-sheet structure formation and silk fibroin self-assembly. However, the effect of subzero temperature treatment was not developed. From these observations, the idea of using freezing treatment for stable scaffold fabrication was studied in this work. The choice of thermal treatment process as a silk scaffold fabrication method was also motivated by its simplicity. The porogen (i.e., ice crystals) formed during freezing can form a pore network, which could be easily removed and does not require an aqueous wash and, thus, does not affect the biological activities of seeded growth factors or cells [46]. Moreover, with the releasing of water, the hydrophobic and hydrophilic blocks of initial silk structure could aggregate to each other and connect into a network, due to their strong intermolecular interactions forming by the removal of the water. One of the key aspects of this study was investigating the effect of thermal treatment on silk fibroin solution, as well as developing engineered 3D architectures based on silk self-assembly. The only solvent used was distilled water, and no cross-linking agent was applied. Moreover, extreme temperature applied (−80°C) is also the same for cell storage, which means cells can be added to sterilized silk solution before being frozen under −80°C to become a cell-material matrix, which could be directly implanted into the body.

The results from this study demonstrated that silk fibroin protein was available to be self-assembled into a scaffold by thermal treatment. Self-assembly occurs in two steps: establishment of the initial structure that occurred at room temperature followed by subsequent growth and elongation [1]. The microscope figure of untreated silk solution confirms the structural progression of fibroin precursors to micelles (Figure 5, day 0) [1]. Once the proteins have been folded into micelles, they were available for self-assembly due to the association of hydrophobic regions, which leads to aggregation into stable nanofilaments (Figure 5) [1]. This elongation process required extensive time and energy to rearrange the hydrophobic and hydrophilic blocks of the protein chains, prepared with different levels of hydrophilic interactions [1]. Following increased time for this rearrangement, interactions between the silk blocks also increased. The sufficient time for silk fibroin's elongation into a stable structure which is investigated by this work shows that the optimized freezing time was 6-7 days.

Another key factor of the process was the thawing rate. The result implied that sudden increment of energy by temperature shocking could break the intermolecular noncovalent interactions, leading to the collapse of the filaments into small fractions or micelles. The experimental design for slow and steady increment of the temperature was used to decrease the thawing rate. Although self-assembly could occur in the freezing process, the sudden increase in temperature, or the energy burst, could break the intermolecular weak bonds among the silk fibers and lead to the collapse of the structure into small fractions. The optimized thawing time was 48 hours, which was available to fabricate stable silk scaffold successfully. Compared to thawing rate and freezing time, freezing temperature did not have an impact effect on the silk self-assembly process. However, the unsystematic morphology of −20°C group indicated that −80°C was a better freezing temperature for the self-assembly process.

The scaffold characterization indicated that the scaffold fabricated by thermal treatment method had high and adjustable pore size, which was easy to control by solution concentration. Pore size is an essential consideration of scaffolds for tissue engineering [47, 48]. If pores are too small, cells cannot migrate into the center of the construct, limiting the diffusion of nutrients and removal of waste products which could lead to necrotic regions within the construct. Conversely, large pore size could decrease the surface area and limit cell attachment. That is the reason why pore size controllability is an important property for scaffold applied for tissue engineering. However, there was not an optimal pore size for cell attachment, proliferation, and migration, due to its heavy dependence on the cell type of concern [49]. It has been reported that pore sizes between 5 and 15 *µ*m are suitable for fibroblast, 20 *µ*m for hepatocytes, and 100 to 400 *µ*m for bone regeneration [50–52]. Moreover, previous studies demonstrated that 100–135 *µ*m is the optimal range for bone growth, greater than 300 *µ*m is essential for vascularization, and smaller than 300 *µ*m could encourage osteochondral ossification [47, 48]. This information and the result from this study implied that 3.6% and 2.4% samples performed as a great candidate for bone regeneration. 32.69% and 22.45% of the pore sizes of 3.6% group and 2.4% group, respectively, are between 100 *µ*m and 135 *µ*m, the most optimal range for bone growth. On the other hand, 6.0% and 4.8% scaffolds were more suitable for skin or soft tissue regeneration (pore size mostly smaller than 100 *μ*m).

The secondary structural composition is one of the most important pieces of information for protein. FT-IR spectra were used to confirm the amide I structural transform of −80ST48 silk scaffold compared to silk solution and alcohol-immersed one. However, due to the overlap of the underlying component bands, mathematical methods were required to analyze specific secondary structure, especially *β*-sheet component. In this work, deconvolution technique was used to indicate the percent of *β*-sheets among other secondary structures to judge the efficiency of the self-assembly process. The result demonstrates the conformation of silk protein into a secondary structure inside the −80ST48 or alcohol-immersed scaffold. Compared to the alcohol-immersed sample, in −80ST48 sample, the percentage of *β*-sheet was relatively decreased. Because the *β*-sheets structure was proven to increase the mechanical strength and toughness of silk scaffold, this result implies that the mechanical strength and toughness of −80ST48 scaffold were lower than of the alcohol-immersed one [24, 33]. The wettability test showed that −80ST48 scaffold had a high water absorption rate, which is another essential factor to cell migration, nutrient flow, oxygen supplement, and waste removal.

The cell's viability, adhesion, and proliferation on the scaffold were used to indicate the biocompatibility and appropriateness of medical application. Because proliferation of most mammalian cell types depends on anchorage, the ideal scaffold must provide a suitable ability for structural template for cells to adhere onto, enabling them to proliferate, differentiate, migrate, and deposit extracellular matrix proteins [53, 54]. In this study, silk scaffold was packed and filled with large quantities of cells in the animal test. However, the distribution of cells in implant scaffolds clearly demonstrated the cell migration into the 3D scaffold, which meant greatly regenerated tissue formation in the interface layer and poor cell attachment in the middle field at 3 weeks after implantation. The problem of distribution of cells throughout scaffolds leading to the lack of uniformity of newly regenerated tissue could be explained by the lack of nutrient diffusion through the scaffold and the slow cell migration rate, due to the poor display of host tissue integration and vascularization [55]. Other research proved that cell only survives in a diffusion distance of 150 to 200 *µ*m from the supplying blood vessel [56, 57].

## 5. Conclusion

In the present study, we focused on roles of thermal temperature in controlling the silk fibroin self-assembly. The factors of thermal treatment were investigated and optimized. The present work also demonstrates the successful preparation of the porous silk fibroin scaffolds by self-assembly process under thermal treatment. The prepared −80ST48 scaffolds were highly porous and had controllable pore size and high water absorption rate. The in vivo result showed the cell attachment and proliferation well on these porous scaffolds confirming the biocompatibility which can be used as an alternative method in various tissue engineering applications.

There are still many challenges in using silk material as a 3D scaffold via the thermal process, such as sterilization of silk solution, unfavorable cellular changes in temperature-increasing rate time, and in vivo nutrient flow. Several further studies should be done before 3D cell-capsuled silk scaffold could be fabricated. However, the work in this study demonstrated the practicality of self-assembly application for silk and silk-based materials.

## Figures and Tables

**Table 1:** The structural conformation ratios in −80ST48, alcohol-immersed, and control samples derived from deconvoluted amide I FT-IR spectra.

	Coefficient of determination (CoD)	*β*-sheet	Random coil/*α*-helix	*β*-turn
−80ST48 scaffold	0.99999	5.80%	63.10%	31.10%
Control	0.99692	0.00%	100%	0%
Alcohol-immersed scaffold	0.99194	20.33%	58.28%	21.39%
